# Apomixis in plant reproduction: a novel perspective on an old dilemma

**DOI:** 10.1007/s00497-013-0222-y

**Published:** 2013-07-14

**Authors:** Gianni Barcaccia, Emidio Albertini

**Affiliations:** 1 Laboratory of Genetics and Genomics, DAFNAE, University of Padova, Campus of Agripolis, Viale dell’Università 16, 35020 Legnaro, Italy; 2 Department of Applied Biology, University of Perugia, Borgo XX Giugno 74, 06121 Perugia, Italy

**Keywords:** Apomixis, Plant reproduction, Hybrids, Seed production

## Abstract

Seed is one of the key factors of crop productivity. Therefore, a comprehension of the mechanisms underlying seed formation in cultivated plants is crucial for the quantitative and qualitative progress of agricultural production. In angiosperms, two pathways of reproduction through seed exist: sexual or amphimictic, and asexual or apomictic; the former is largely exploited by seed companies for breeding new varieties, whereas the latter is receiving continuously increasing attention from both scientific and industrial sectors in basic research projects. If apomixis is engineered into sexual crops in a controlled manner, its impact on agriculture will be broad and profound. In fact, apomixis will allow clonal seed production and thus enable efficient and consistent yields of high-quality seeds, fruits, and vegetables at lower costs. The development of apomixis technology is expected to have a revolutionary impact on agricultural and food production by reducing cost and breeding time, and avoiding the complications that are typical of sexual reproduction (e.g., incompatibility barriers) and vegetative propagation (e.g., viral transfer). However, the development of apomixis technology in agriculture requires a deeper knowledge of the mechanisms that regulate reproductive development in plants. This knowledge is a necessary prerequisite to understanding the genetic control of the apomictic process and its deviations from the sexual process. Our molecular understanding of apomixis will be greatly advanced when genes that are specifically or differentially expressed during embryo and embryo sac formation are discovered. In our review, we report the main findings on this subject by examining two approaches: i) analysis of the apomictic process in natural apomictic species to search for genes controlling apomixis and ii) analysis of gene mutations resembling apomixis or its components in species that normally reproduce sexually. In fact, our opinion is that a novel perspective on this old dilemma pertaining to the molecular control of apomixis can emerge from a cross-check among candidate genes in natural apomicts and a high-throughput analysis of sexual mutants.

## Introduction

One of the greatest success stories in modern agriculture has been the tremendous yield increase achieved by coupling high-yield varieties with high-input agronomic systems, creating the so-called Green Revolution. Approximately one-third of the world’s seed supply comes from the commercial seed market, another one-third is provided by publicly funded institutions, and the seed saved by farmers accounts for the remainder.

Over the centuries, crop plants have followed the general pattern of introduction, selection, and hybridization. Crop introduction has been crucial for agriculture because many of the world’s crops are produced outside their region of domestication. Once introgressed, selection and breeding strategies have led to the development of new cultivars with improved yield and adaptation. Plant breeders are working to extend the Green Revolution by intensifying selection, developing more hybrid varieties in more crops, and increasing the range of plant functions through mutation and transgenic breeding. Hence, plant breeding will continue to play a crucial role in crop improvement because the needs are many, the techniques are expanding, the new genetic combinations are limitless, and the successes of the past illuminate the potential of the future. In outcrossing species, alleles disseminate in the offspring; thus, the optimal genotype is lost together with the desired trait. Exact copies of a superior genotype can be made via vegetative propagation; however, this technique is usually not applicable to annual crops such as maize, rice, and wheat. The fixation of a given genotype occurs naturally in species that exhibit an asexual type of seed production termed apomixis. This trait by itself is highly valuable for agriculture; however, despite many efforts, it has not been possible to introduce apomixis into modern domesticated crop species.

As a reproductive strategy for cloning plants via seeds, apomixis is a highly desirable trait in modern agriculture. In fact, apomixis results in offspring that are exact genetic replices of the female parent because embryos are derived from the parthenogenic development of apomeiotic egg cells (for reviews on apomixis, see Bicknell and Koltunow 2004; Ozias-Akins 2006; Albertini et al. 2010; Pupilli and Barcaccia 2012; Koltunow et al. 2013). From an evolutionary point of view, apomixis may be regarded as a consequence of sexual failure rather than as a recipe for clonal success (Silvertown 2008).

Introgression of apomixis from wild relatives into crop species and transformation of sexual genotypes into apomictically reproducing genotypes are long-held goals of plant breeding. Breeders believe that the introduction of apomixis into agronomically important crops will have revolutionary implications for agriculture. The potential benefits of harnessing apomixis are many and vary from full exploitation of heterosis by reseeding the best hybrids to clonal propagation of the superior genotypes in seed-propagated outcrossing crops. The impact of apomictic crops in agriculture would be massive in both developed and developing countries. Unfortunately, barring a few exceptions in some forage grasses and fruit trees, apomixis is not a common feature among crop species.

**Fig. 1:**
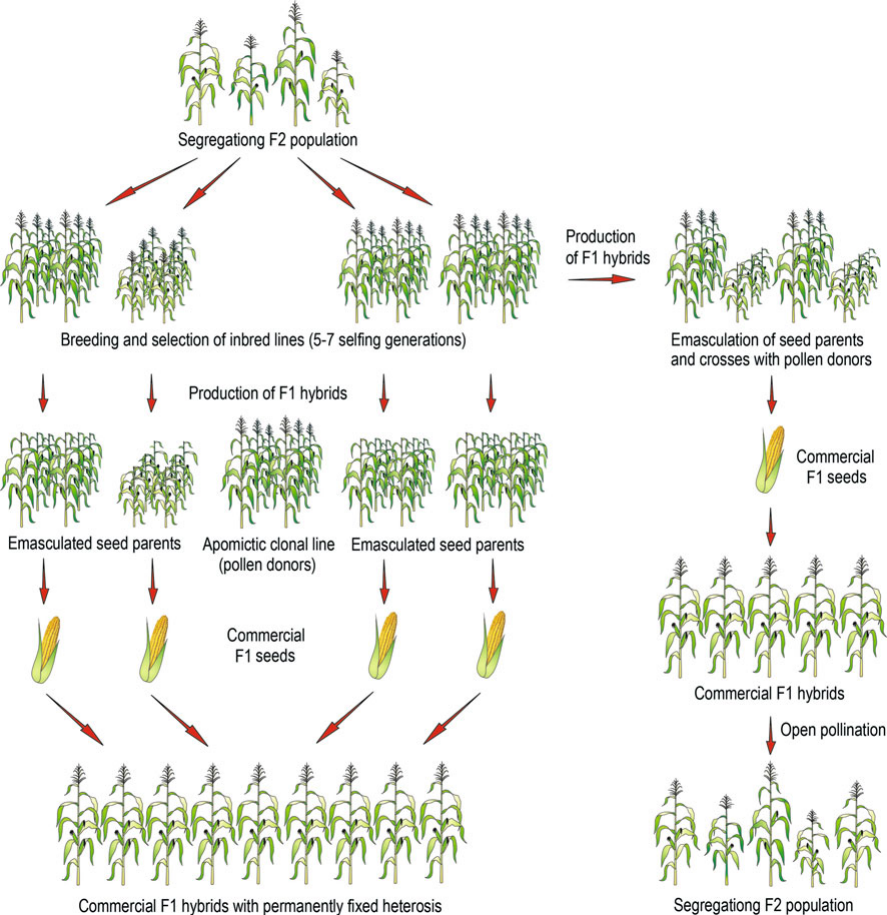
Comparison between conventional and apomixis-mediated methods for breeding F_1_ hybrid varieties. In traditional breeding, within a segregating population (e.g., F_2_ population) some genotypes are selected and after some generation of selfing followed by phenotypic selection, tested for their specific combining ability in order to be used as parental lines for the constitution of heterotic F_1_ hybrid seeds. The best performing inbred lines are selected, multiplied in isolated fields, and crossed in pairwise combinations to obtain uniform, vigorous, and high-yield F_1_ hybrids. This scheme, however, requires a series of actions: the two inbred lines must be kept pure and multiplied in separate fields. Then, to obtain the hybrid seed, it is necessary to establish a dedicated field where about one quarter of the plants is used as pollinator (i.e., pollen donor inbred) and on the remaining plants (i.e., seed parent inbred) the hybrid F_1_ seeds will be harvested. Farmers cannot re-use seeds collected from F_1_ hybrids as these seeds will give rise to highly variable populations because of genetic segregation and recombination. Using apomictic lines, however, the situation would be much simpler. Once superior inbred lines to be used as seed parent are selected, they can be crossed with clonal lines as pollen donors carrying the gene for apomixis, in order to obtain F_1_ hybrid seeds sharing a highly heterozygous genotype. From this moment on, each F_1_ hybrid variety can be maintained for several generations with permanently fixed heterosis

The fixation of hybrid vigor through apomixis is a desirable objective for breeders and farmers alike and is expected to have a revolutionary impact on food and agriculture production. The stabilization of heterozygous genotypes via apomixis would make breeding programs faster and cheaper (Fig. 1). The impact of apomictic crops in agriculture would be comparable to, or even greater than, the impact of the Green Revolution, especially in Third World countries (Vielle-Calzada et al. 1996; Pupilli and Barcaccia 2012). In fact, it has been estimated that the use of apomixis technology in the production of hybrid rice alone could provide benefits exceeding 1,800 million Euros per year (Spillane et al. 2004; Albertini et al. 2010). Apomixis technology could also provide benefits for clonally propagated crops. Clonal crop yields are limited by pathogens (mainly viral and endophytic), which accumulate over successive rounds of vegetative propagation and seriously limit the yield and exchange of germplasms between countries. The use of apomixis technology in these crops would provide the additional option and benefit of propagation via clonal seeds and thus generating disease-free material that can be more easily stored and transported. The use of apomictic seed as an alternative to vegetative propagules would provide similar benefits (e.g., lower costs and higher yields) over the current use of true seed of such crops. For example, apomixis technology could make true potato seeds a more attractive option for potato breeders and cultivators and would return benefits to growers of as much as 2.3 billion Euros per year (Spillane et al. 2004).

The development of apomixis technology in agriculture will require a deeper knowledge of the mechanisms regulating reproductive development in plants. Our molecular understanding of apomixis would be greatly increased if genes that are specifically or differentially expressed during the formation of the embryo and embryo sac could be identified. Over the last two decades, many scientists have speculated about the isolation of gene/s controlling key steps of the apomictic pathway, and many papers have postulated the production of engineered plants exhibiting apomictic-like phenotypes. In fact, none of the major crop plants have been bred for apomixis, and only some features of apomixis have been genetically engineered in model species. Consequently, even in the era of genomics, achieving an understanding of the genetic control and molecular regulation of apomixis appears much more complicated than expected. Large amounts of cytological and ecological information, along with genetic and molecular data, have been collected mainly from model species (i.e., *Boechera holboellii*, *Hieracium* spp., *Hypericum perforatum*, *Paspalum* spp., *Poa pratensis*, *Ranunculus* spp., and *Taraxacum officinale*) and have often been tested in *Arabidopsis thaliana* (*Arabidopsis*) to elucidate the mechanisms of apomeiosis, parthenogenesis, and apomixis. Several genes involved in the formation of unreduced embryo sacs and egg cells, in addition to genes responsible for the autonomous development of the embryo and endosperm, have been cloned and characterized; however, none of these genes are capable of miming the apomictic pathway as a whole in crop plants. Hence, after two decades of substantial studies conducted in several laboratories and model plants, the asexual reproductive strategy termed “gametophytic apomixis” by Nogler (1984) still appears to be an unsolved puzzle. As a result, seed companies have lost interest in this research, and it has been difficult to acquire funds for conducting research on apomixis.

Currently, novel views and original concepts are emerging from the fog, including a link between apomixis and gene-specific silencing mechanisms (likely based on chromatin remodeling factors or trans-acting and heterochromatic interfering RNAs involved in both transcriptional and post-transcriptional gene regulation) and the parallel between the Y chromosome and apomixis-bearing chromosomes from the most primitive to the most advanced in evolutionary terms (comparative genomic analyses revealed common features such as few recombination events, accumulation of transposable elements, and degeneration of genes). More recently, merging lines of evidence regarding the role of auxin in cell fate specification of the embryo sac and egg cell development have been reported in *Arabidopsis*.

## Mechanisms of apomixis

Because apomictic reproduction entails the development of an embryo from a cell with a somatic chromosome number, there are several ways to produce embryos of apomictic origin. The simplest pathway avoids the production of an embryo sac, and the maternal embryo originates from one or more somatic cells of the ovule. Among the agriculturally important species, adventitious embryony (i.e., sporophytic apomixis) has been noted in mango (*Mangifera indica*), several *Citrus* species, and orchids. The most comprehensive treatise on adventitious embryony has been published by Naumova (1992).

When the maternal embryo originates from a diploid egg cell differentiated in an unreduced embryo sac, the apomictic pathway is referred to as gametophytic apomixis (Nogler 1984).

In gametophytic apomixis, the unreduced embryo sac may arise from a somatic nucellar cell that acquires the developmental program of a functional megaspore, a mechanism referred to as apospory. Alternatively, if the embryo sac forms from a megaspore mother cell with suppressed or modified meiosis, the pathway is referred to as diplospory. It is worth emphasizing that apomictic plants may or may not change meiosis itself, but in any case they do activate the gametic cell fate either in a somatic cell (apospory) or in an unreduced megaspore (diplospory) as surrogate for meiotic products (Albertini and Barcaccia 2007). Once 2*n* female gametophytes and gametes are formed (apomeiosis), they subsequently undergo embryogenesis autonomously without fertilization by a male gamete (somatic parthenogenesis). Endosperm formation may be fertilization-independent (autonomous endosperm) or may require fertilization (pseudogamous endosperm). Among others, apospory has been reported in *Beta*, *Brachiaria*, *Cenchrus*, *Chloris*, *Compositae*, *Eriochloa*, *Heteropogon*, *Hieracium*, *Hyparrhenia*, *Hypericum*, *Panicum*, *Paspalum*, *Pennisetum*, *Poaceae*, *Ranunculus*, *Sorghum*, *Themeda*, and *Urochloa*, whereas diplospory has been noted in *Agropyrum*, *Allium*, *Antennaria*, *Boechera* (formerly *Arabis*), *Datura*, *Eragrostis*, *Erigeron*, *Eupatorium*, *Ixeris*, *Parthenium*, *Paspalum*, *Poa*, *Taraxacum*, and *Tripsacum* (Table 1).

**Table 1:** Basic information on inheritance models, genetic recombination potentials, and molecular mapping studies of apospory and diplospory in apomictic species

Apomixis system Species	Endosperm development	Suppression of recombination	References for genetic mapping and inheritance	Candidate genes	References for candidate genes
Apospory					
*Brachiaria brizantha*	Pseudogamous	Yes	do Valle and Savidan (1996), Pessino et al. (1997, 1998)	*RPS8*-*RPS15a*-*RPL41* *Sti1*; *Helic*	Lacerda et al. (2013), Silveira et al. (2012)
*C. ciliaris*	Pseudogamous	No	Sherwood et al. (1994), Roche et al. (1999), Jessup et al. (2002), Dwivedi et al. (2007), Conner et al. (2013)	*BBM*-*like*	Conner et al. (2008)
*Hieracium* spp.	Autonomous	No	Bicknell et al. (2000), Catanach et al. (2006), Koltunow et al. (2011), Tucker et al. (2012)	*HFIE*	Rodrigues et al. (2008, 2010a)
*H. perforatum*	Pseudogamous	No	Matzk et al. (2001), Barcaccia et al. (2006, 2007)	*ARIADNE7* (*ARI7*) *HAPPY* *locus*	Schallau et al. (2010), Galla et al. (personal communication)
*P. maximum*	Pseudogamous	No	Ebina et al. (2005), Kaushal et al. (2008)	*ASG*-*1*	Chen et al. (1999, 2005)
*P. notatum*	Pseudogamous	Yes	Martinez et al. (2003), Stein et al. (2007)	*LORELEI*	Felitti et al. (2011)
*P. simplex*	Pseudogamous	Yes	Pupilli et al. (2001), Labombarda et al. (2002)	Unspecified (*ACR*)	Calderini et al. (2012), Pupilli (personal communication)
*P. ciliare*	Pseudogamous	Yes	Jessup et al. (2002, 2003)	*Pca21*, *Pca24*	Singh et al. (2007)
*P. squamulatum*	Pseudogamous	Yes	Ozias-Akins et al. (1998), Goel et al. (2006), Huo et al. (2009)	*BBM*-*like*	Conner et al. (2008)
*P. pratensis*	Pseudogamous	No	Barcaccia et al. (1998), Albertini et al. (2001), Matzk et al. (2005)	*SERK*, *APOSTART1*-*2*	Albertini et al. (2004, 2005)
*Ranunculus auricomus*	Pseudogamous	No	Nogler (1984, 1995)	ND	
Diplospory					
*Boechera* spp.	Pseudogamous	Yes	Schranz et al. (2006), Lovell et al. (2013)	*APOLLO*	Corral et al. (2013)
*Erigeron annuus*	Autonomous	No	Noyes and Rieseberg (2000), Noyes et al. (2007)	ND	
*T. officinale*	Autonomous	No	Tas and van Dijk (1999), van Dijk et al. (1999, 2003), Vijverberg et al. (2004, 2010)	ND	
*T. dactyloides*	Pseudogamous	Yes	Leblanc et al. (1995), Grimanelli et al. (1998)	ND	

Candidate genes for apomixis are also given along with references

## Inheritance of apomixis: genetic control and recombination potentials

In both aposporic and diplosporic species, robust models have been postulated and eventually validated for inspecting the genetic basis of apomixis and its components (i.e., apomeiosis and parthenogenesis). The inheritance fundamentals include a divergence in the number of genes, gene functions, and relationships among alleles, as well as dominance of apomixis over sexuality (Asker and Jerling 1992; Carman 1997; Savidan 2000; Grimanelli et al. 2001; Koltunow and Grossniklaus 2003). Genetic analysis in several species has consistently demonstrated that a simple inheritance system involving a few Mendelian genes controls the expression of apomixis or its components. In contrast, molecular and cytogenetic analyses of chromosomal region(s) carrying the determinants of apomixis in several species have revealed a complex genetic control mechanism that is likely based on a system of polygenes in addition to mechanisms involving a lack of recombination, trans-acting elements for gamete elimination, supernumerary chromatin structures, and DNA rearrangements (see a review by Pupilli and Barcaccia 2012 and references therein).

Currently, gametophytic apomixis is thought to rely on three genetically independent Mendelian loci, each exerting control over a key developmental component, including apomeiotic megaspores, parthenogenic unreduced egg cells, and modified endosperms (Grossniklaus et al. 2001a; Koltunow and Grossniklaus 2003; Bicknell and Koltunow 2004; Vijverberg and van Dijk 2007; see also a review by Albertini et al. 2010 and references therein). A single regulatory gene was originally proposed as being sufficient for promoting apomixis (Peacock 1992). Although simple genetic control seems to support this hypothesis, molecular evidence suggests that a more complex inheritance system directs the entire process of apomixis. In some species, linkage groups typically transmitted with apomixis contain large blocks of sequence that lack genetic recombination between molecular markers, leading to speculation that adapted gene complexes within supernumerary chromatin might be required for the expression of apomixis (Ozias-Akins et al. 1998; Roche et al. 2001; Akiyama et al. 2004). A close relationship between apomictic mechanisms and heterochromatic regions of the genome that are rich in retrotransposons has raised the intriguing possibility that DNA structure and/or RNA interference could play a role in regulating the expression of apomixis-related genes (Pupilli and Barcaccia 2012). A well-characterized class of small regulatory RNAs that are widespread in eukaryotes appears to regulate gamete function and fertilization in plants by altering gene expression through post-transcriptional gene silencing, translational inhibition, and heterochromatin modification (Ron et al. 2010).

Increasing experimental evidence suggests that genetic recombination can either be suppressed or allowed in chromosomal regions surrounding the master locus for apomixis depending on the evolutionary pathway of the genome. This is a phenomenon that has been well documented for plant chromosomes carrying sex-determining genes (Vyskot and Hobza 2004). From an evolutionary point of view, we expect that relatively young and simple genetic systems of apomixis determination should include a narrow euchromatic region where genetic recombination between apomeiosis and parthenogenesis loci, and their linked genes, is possible (Table 1), as in *Poa* (Barcaccia et al. 2000; Albertini et al. 2001), *Taraxacum* (van Dijk and Bakx-Schotman 2004), *Hypericum* (Schallau et al. 2010), *Erigeron* (Noyes and Rieseberg 2000), *Hieracium* (Catanach et al. 2006), and *Panicum maximum* (Kaushal et al. 2008). In contrast, a degenerate heterochromatic block carrying apomixis factors should represent evolutionarily advanced genetic systems of apomixis determination with large non-recombining regions surrounding the apomixis locus (Table 1) as in *Pennisetum*/*Cenchrus* (Ozias-Akins et al. 1998; Roche et al. 1999), *Brachiaria* (Pessino et al. 1998), *Paspalum* (Labombarda et al. 2002; Stein et al. 2007; Podio et al. 2012), and *Tripsacum* (Grimanelli et al. 1998). Just recently, Conner et al. (2013) found recombination between apospory and parthenogenesis loci in *C. ciliaris* (Table 1). Based on the experimental data collected thus far, Pupilli and Barcaccia (2012) have recently hypothesized that a relatively simple genetic system controls apomixis in terms of the number of genes involved in the expression of its components (i.e., genes controlling apomeiosis and parthenogenesis and eventually autonomous endosperm development). However, elements within the chromosome block carrying the apomixis genes (e.g., transposable elements, repetitive elements, and pseudogenes) make it a complex genetic system, with loci that vary from elementary and primitive to evolutionarily advanced. According to recent findings, the first type reflects a chromosome pair showing tightly linked genetic determinants for apomixis in a narrow euchromatic region where genetic recombination is not suppressed, whereas the other type includes a chromosome pair that possesses a degenerate gene block with a large non-recombining region surrounding the apomixis locus (for details see Pupilli and Barcaccia 2012).

## Searching for genes controlling apomixis in natural apomicts

Although many years of descriptive studies have provided a solid documentation of the types of apomictic processes that occur in a wide variety of plant species, molecular studies aimed at understanding the basis of apomixis have failed to adequately elucidate its central mystery, partly because the majority of apomicts do not constitute agriculturally important crops and, with a few exceptions (e.g., *Tripsacum* and maize), do not have agriculturally important relatives (Bicknell and Koltunow 2004; Albertini et al. 2010). An early theory regarding genetic control of apomixis proposed that the trait is regulated by “a delicate gene balance” (Muntzing 1940) of recessive genes and that this balance might be disturbed after crosses. Currently, basic inheritance is usually thought to depend on a single master regulatory gene or a few dominant key genes, which allow a megaspore mother cell or a somatic nucellar cell to form an embryo sac without meiotic reduction and an embryo to develop from an unreduced egg cell without fertilization (Asker and Jerling 1992; Koltunow et al. 1995; Savidan 2000; Grossniklaus et al. 2001b). Once apomictic genes initiate embryo development and the initial cell forms and divides, the genes controlling embryo cell formation and patterning are most likely the same as those required for sexual embryo development. Whether the products of apomictic genes are proteins that are not produced in sexually reproducing plants (i.e., gain of function) or proteins that normally function to initiate events in sexual reproduction but have become altered with respect to their activity or spatial and temporal distribution during development (i.e., loss of function) is still not well understood. Currently, a number of researchers support the hypothesis that zygotic embryogenesis and apomictic parthenogenesis follow similar pathways during embryo and seed production (Bicknell and Koltunow 2004; Albertini et al. 2004; Sharbel et al. 2010). Specific genes are activated, modulated, or silenced in the primary steps of plant reproduction to ensure that functioning embryo sacs develop from meiotic spores and/or apomictic cells. Because additional genes may be specifically or differentially expressed in sexually versus apomictically reproducing plants, and these genes may operate during embryo development, we would be better equipped to understand apomixis if the genes responsible for controlling specific and differential expression during embryo and embryo sac formation could be identified.

Some scientists believe that apomixis is controlled by specific genes encoding new proteins with a novel initiating function not observed in sexually reproducing plants, and these scientists have performed experiments based on either differential display or subtractive hybridization of a single flower stage. This led to the identification of a number of candidate genes. For example, in *Brachiaria* species, among the 12 candidates isolated by Leblanc et al. (1997), only two proved to be specifically expressed in mature ovaries containing unreduced (aposporic) embryo sacs. Instead of genes specifically expressed either in apomictic or sexual genotypes, Rodrigues et al. (2003) searched for candidates expressed in both genotypes of the same species and identified 11 genes that were differentially expressed between apomictic and sexual genotypes. In *Paspalum notatum*, three distinct gene transcripts showed differential expression between apomictic and sexual F_1_ individuals after apospory initiation in flowers (Pessino et al. 2001). An additional 65 genes that were differentially expressed between apomictic and sexual genotypes at the meiotic stage were identified by Laspina et al. (2008). A large subset of these candidates mapped in silico to a genomic region on rice chromosome 2 that was previously associated with apospory (Pessino et al. 1998; Pupilli et al. 2004), and one of these genes showed high similarity to *lorelei*, a gene associated with male gamete delivery to the egg cell in *Arabidopsis* (Felitti et al. 2011).

Candidate genes specifically expressed in either apomictic or sexual ovules were also identified in *P. maximum* (Chen et al. 1999, 2005; Yamada-Akiyama et al. 2009) and *Pennisetum ciliare* (Vielle-Calzada et al. 1996). Additional genes differentially expressed between apomictic and sexual samples were isolated in *P. maximum* (Yamada-Akiyama et al. 2009), *P. ciliare* (Vielle-Calzada et al. 1996; Singh et al. 2007), *Hieracium pilosella* (Guerin et al. 2000), and *Eragrostis curvula* (Cervigni et al. 2008; Selva et al. 2012).

Although a large number of candidate genes exhibiting differences in spatial and temporal expression levels and patterns have been identified with these approaches, both their function and their involvement in the control of apomixis remain largely speculative (Ozias-Akins 2006). Moreover, although negative in context, these studies added support to the second theory, which proposes that apomixis is controlled by proteins that normally function to initiate events in sexual reproduction but may be altered with respect to their activity or spatial and temporal distribution during development (Bicknell and Koltunow 2004). One of the first supporters of this hypothesis was Carman (1997), who suggested that apomixis is a result of the deregulation of sex-related genes with respect to spatial and temporal expression as a consequence of their heterochronic expression due to hybridization (Singh et al. 2007; Sharbel et al. 2009; reviewed by Albertini et al. 2010). Research carried out in species that reproduce through distinct pathways seemed to confirm that apomixis relies upon either spatial or temporal misexpression of genes acting during female sexual reproduction (Grimanelli et al. 2003; Tucker et al. 2003; Albertini et al. 2004; Curtis and Grossniklaus 2007; Sharbel et al. 2009). Attempts to isolate asynchronously regulated genes were carried out by comparing the transcriptional profiles of apomictic and sexual ovules over several developmental stages in several species because careful staging was thought to be critical for the interpretation of the results, particularly if misexpression, rather than unique expression, was responsible for the switch in reproduction mode (Ozias-Akins 2006). Recently, careful staging of ovary development has led to the identification of differentially expressed transcripts in *P. pratensis* (Albertini et al. 2004, 2005; Marconi et al. 2013), *B. holboellii* (Sharbel et al. 2009, 2010), *Paspalum simplex* (Polegri et al. 2010), and *H. perforatum* (Galla and Barcaccia 2012; Galla et al., personal communication).

**Fig. 2:**
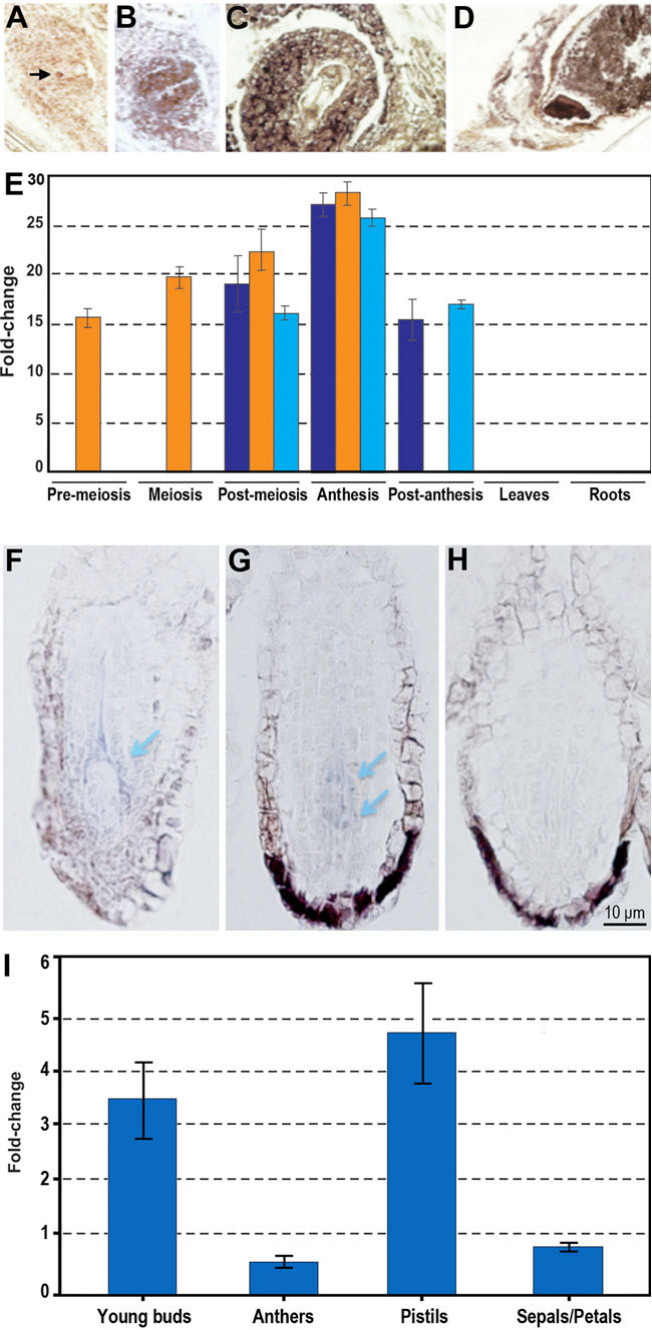
Expression data related to candidate genes for apomixis. Gene expression patterns and levels of *APOSTART6* in *P. pratensis* and *ARIADNE7* in *H. perforatum* as assessed by in situ hybridization and real-time RT-PCR analysis. **a**–**d** *APOSTART6* expression patterns in longitudinal sections of *P. pratensis* ovaries: signal is present in one or more nucellar cells (*arrow*) within the ovule of apomictic genotypes (**a**) and in the megaspore mother cell in sexual genotypes (data not shown, for details see Albertini et al. 2005). Signal is then present during embryo sac development (**b**, **c**) and embryo development (**d**). **e** Expression patterns and level of transcripts encoded by *APOSTART6* in apomictic (*dark blue*), sexual (*red*), and parthenogenic recombinant (*light blue*) genotypes of *P. pratensis* (for details see Marconi et al. 2013). Delay of expression in apomictic and parthenogenetic genotypes suggests an involvement of *APOSTART6* in parthenogenesis. **f**, **g** Longitudinal sections of *H. perforatum* ovules at the stage of female meiosis showing hybridization signals of *ARIADNE7* transcripts (*arrows*) in correspondence with nucellar tissues next to megaspores; **h** negative control (courtesy of Giulio Galla, University of Padova). **i** Expression levels of the *ARIADNE7* transcripts in young buds, anthers, pistils, and sepals and petals: this gene was found preferentially expressed in pistils and young buds of apomictic genotypes. Specificity of expression domain in apomictic and aposporic genotypes suggests an involvement of *ARIADNE7* in apospory

In *P. pratensis*, Albertini et al. (2004) isolated as many as 179 fragments that were differentially expressed between apomictic and sexual genotypes. Importantly, most of the transcripts were not specifically associated with apomictic or sexual genotypes; instead, expression was differentially modulated or quantitatively different (Albertini et al. 2004, 2005), supporting the hypothesis that apomixis may result from a deregulated sexual pathway. In particular, *PpSERK* and *APOSTART* (Table 1) were characterized in detail (Albertini et al. 2005) and were thought to be involved in cell-to-cell signaling and hormone trafficking. The authors proposed that *PpSERK* activation in nucellar cells of apomictic genotypes is the switch that triggers embryo sac development and could redirect signaling gene products to compartments other than their typical ones. The SERK-mediated signaling pathway may interact with the auxin/hormonal pathway controlled by APOSTART. Indeed, based on its temporal and spatial expression patterns, *APOSTART* is potentially associated with apomixis, and its transcripts are detectable specifically in aposporic initials and embryo sacs. Additionally, gene expression studies of gene members revealed a delay of *APOSTART6* expression in apomictic and parthenogenic *P. prantensis* genotypes (Fig. 2, panels a–e), supporting an involvement of this allele in parthenogenesis (Marconi et al. 2013). Overall, the accumulated data suggest that APOSTART may be related to programmed cell death (PCD) that is involved in the non-functional megaspore and nucellar cell degeneration events that permit enlargement of maturing embryo sacs. Functional characterization of the *Arabidopsis APOSTART1* gene (*AtAPO1*) showed that it is expressed in mature embryo sacs and developing embryos. APOSTART1/APOSTART2 double mutants seem to confirm an involvement of this gene in embryo/seed development (Albertini et al. unpublished data).

More recently, Sharbel et al. (2009, 2010) utilized a high-throughput differential display approach to study naturally occurring quantitative variations in gene expression between ovules of apomictic and sexual *B. holboellii* genotypes and identified 543 genes exhibiting a developmental shift in expression between the sexual and apomeiotic ovules. A carefully devised experiment was also undertaken by Polegri et al. (2010), leading to the identification of a set of genes in *P. simplex* with stage- and/or phenotype-specific expression. In particular, a class of alleles that showed a characteristic specificity of expression was fully linked to apomixis based on mapping data.

In *H. perforatum*, a differential display analysis of sporogenesis and gametogenesis led to the isolation of several transcripts specifically expressed in the pistils of a highly apomictic ecotype, including an EST showing similarity to a gene coding for an ATPase RNA helicase responsible for an embryo defective phenotype in *Arabidopsis* (*MEE29*, maternal effect embryo). This gene, termed *HpMEE29*-like, was differentially expressed between aposporic and meiotic *H. perforatum* plants (Barcaccia et al. 2007). More recently, Galla and Barcaccia (2012) adopted high-throughput 454 technology to sequence the entire *Hypericum* flower transcriptome using single verticils collected from apomictic and sexual genotypes. Computational procedures were used to assemble and annotate more than 25,000 transcripts exclusively from anthers and carpels at different developmental stages. Galla et al. (2013) identified dozens of genes related to sporogenesis and gametogenesis, with particular reference to the formation of embryo sacs, embryos, and seeds. Interestingly, some of the transcripts showed sequence homology with candidate genes for apomixis that were cloned in apomictic species and mutants, including components of the HAPPY locus (Schallau et al. 2010). Notably, many small transcripts of genes specifically expressed in apomictic genotypes exhibited high similarity to microRNA precursors that target specific transcription factors. In particular, the most represented and conserved families were miR156, miR166, miR390, miR394, miR396, and miR414, which have dozens of potential target genes with a wide range of molecular functions including metabolism, response to stress, flower development, and plant reproduction Galla et al. (2013).

Different approaches were chosen in *Pennisetum* *squamulatum* (Conner et al. 2008) and *H. perforatum* (Schallau et al. 2010). In *P.* *squamulatum*, by sequencing an apospory-specific genomic region (ASGR), Conner et al. (2008) isolated a gene sharing similarity with *BABY BOOM* (*BBM*), which was named *ASGR*–*BBM* (Table 1). This gene encodes a protein containing two AP2 domains that are 96 % similar to the AP2 regions of *BnBBM* (outside the AP2 domains, the similarity of *ASGR*-*BBM* to *BnBBM* declines significantly to 35 and 27 % in the upstream and downstream regions, respectively). In *H. perforatum*, Schallau et al. (2010) screened genomic clones using an apospory-linked SCAR marker as probe and identified a 142 kb BAC clone containing a gene homologous to *Arabidopsis* *ARIADNE7* (*ARI7*, Table 1), which is annotated as a nucleic acid binding protein. In particular, both aposporic- and sexual-specific *HpARI7* alleles were found co-expressed in the pistils at different developmental stages, whereas the gene product of the apomictic allele was specifically expressed in pistils of the apomictically reproducing individuals (Schallau et al. 2010). More recently, Galla et al. (personal communication) demonstrated that *HpARI7* gene is preferentially expressed in pistils at different developmental stages and that *HpARI7* transcripts are specifically detectable in nucellar tissues of the ovule next to megaspores in apomictic *H. perforatum* genotypes (Fig. 2, panels f–i). Specificity of the expression domain in apomictic and aposporic genotypes suggests an involvement of this gene belonging to the HAPPY locus in apospory.

## Functional analysis of genes miming apomixis in sexual model plants

If it is true that apomixis is a consequence of sexual failure, rather than a means for clonal success, from an evolutionary point of view (Silvertown 2008), it is also true that apomixis, as a biological process of seed formation, represents an altered form of sexuality rather than a new developmental program (Koltunow and Grossniklaus 2003). Carman (1997) hypothesized that apomixis is a result of the spatial and temporal deregulation of sex-related gene expression as a consequence of heterochronic expression due to hybridization. This hypothesis was confirmed experimentally by cytological observations in *Tripsacum* (Grimanelli et al. 2003) and more recently by transcriptome analyses in *Boechera* (Sharbel et al. 2009, 2010). Additionally, Tucker et al. (2003) provided evidence that marker genes related to embryo sac development are similarly expressed in sexual and apomictic *Hieracium* genotypes.

The idea that apomixis is an altered form of sexuality that results from temporal and spatial alterations in the action of the sexual program suggests that a synthetic apomixis system using variant alleles of genes isolated from sexual model species, such as *Arabidopsis*, can be developed (Chaudhury and Peacock 1993). By identifying and combining the genes involved in apomixis, the assembly of an asexual system of seed formation in a sexual plant should be possible. In particular, some of the genes isolated and characterized from sexual species may have roles in apomixis (Table 2). For example, the recent findings regarding the molecular mechanisms controlling embryo sac development, fertilization, and endosperm development may be useful for determining genetic links with apomeiosis, parthenogenesis, and autonomous or pseudogamous endosperm development. It is believed that basic structural and functional analyses of these candidate genes are crucial for engineering apomixis in sexual crops.

**Table 2:** General information on the mutants miming apomictic components and genes showing apomictic phenotypes in sexual model plants along with references

Plant phenotype	Gene/mutant	Species	Gene product/molecular function	References
Apomeiosis				
Multiple archesporial cells	*MAC1*	Maize	n.d.	Sheridan et al. (1996)
	*MSP1*	Rice	LRR receptor-like kinase	Nonomura et al. (2003)
	*TDL1A*	Rice	Small extracellular protein (unknown function)	Zhao et al. (2008)
Diplospory-like (switch/turning meiosis into mitosis)	*DYAD*/*SWITCH* (*SWI*)	Arabidopsis	Meiosis-specific chromatin-associated protein	Ravi et al. (2008), Pawlowski et al. (2009)
	*MiMe* (*SPO11*-*1*, *REC8* or *SYN1* and *OSD1* or *TAM1*)	Arabidopsis	Meiosis-specific (synaptic) proteins and cell cycle-progression proteins	d’Erfurth et al. (2009), 2010)
Diplospory-like (restitutional meiosis)	*Elomgate* (*el1*)	Maize	Putative chromatin remodeling factor	Rhoades and Dempsey (1966), Grimanelli et al. (2003)
	*AGO104*	Maize	ARGONAUTE protein catalytic component of the RNA-induced protein complex of gene silencing	Singh et al. (2011)
	*MOB1*	Alfalfa Arabidopsis	Protein involved in cell proliferation, cell death and cell polarity	Citterio et al. (2005), Vitulo et al. (2007), Galla et al. (2010)
	*DMT102* and *DMT103*		DNA methyl-transferases	Garcia-Aguilar et al. (2010)
Apospory-like (aposporic initials and embryo sacs)	*AGO9*	Arabidopsis	ARGONAUTE protein (see above)	Olmedo-Monfil et al. (2010)
	*RDR6*	Arabidopsis	RNA-dependent RNA polymerase	
	*SGS3*	Arabidopsis	RNA binding protein suppressor of gene silencing	
	*MNEME* (*MEM*)	Arabidopsis	DEAD/DEAH-box RNA helicase	Schmidt et al. (2011)
Parthenogenesis/embryogenesis				
Haploid parthenogenesis and facultative pseudogamy	*Haploid parthenogenesis* (*HAP*)	Barley	n.d.	Hagberg and Hagberg (1980)
Autonomous seed development, including parthenogenesis	*MSI1*	Arabidopsis	WD40 domain protein; part of polycomb group complex (PRC2)	Guitton and Berger (2005), Ingouff et al. (2005)
Haploid parthenogenesis and polyembryony	*Indeterminate gametophyte* (*IG1*)	Maize	LOB domain protein DNA-binding transcription factors	Evans (2007)
Embryogenesis (embryo development)	*Leafy cotyledon* (*LEC1*)	Arabidopsis	CCAAT box-binding factor (HAP3 subunit)	Lotan et al. (1998)
	*SERK1*	Arabidopsis	LRR receptor-like kinase	Hecht et al. (2001)
	*BABY BOOM* (*BBM1*)	Arabidopsis and Brassica	AP2 domain transcription factor	Boutilier et al. (2002)
Endosperm development				
Autonomous endosperm development	*MEDEA* (*MEA*)	Arabidopsis	SET domain Polycomb protein	Grossniklaus et al. (1998)
	*FIE*	Arabidopsis	WD domain Polycomb protein	Ohad et al. (1999)
	*FIS2*	Arabidopsis	C_2_H_2_ zinc-finger protein part of polycomb group complex	Luo et al. (1999)
	*MSI1*	Arabidopsis	Component of the MEA/FIE Polycomb group complex	Köhler et al. (2003), Guitton and Berger (2005), Ingouff et al. (2005)

Genes that are expressed during embryo sac development, and thus putatively involved in differentiating sexual from apomictic pathways, are of particular interest. An embryo sac develops through megaspore mother cell (MMC) differentiation, meiosis, determination of the functional megaspore, and embryo and endosperm development. Because meiosis is either completely bypassed (apospory) or extremely altered (diplospory) in apomixis, genes related to female sporogenesis are thought to be more specifically involved in diplosporic apomixis, and genes involved in embryo sac cell identity are presumably crucial for aposporic apomixis. Overall, genes associated with female gametogenesis and egg cell development are likely shared between sexual and apomictic pathways (for details on these aspects see reviews by Tucker et al. 2003; Koltunow and Grossniklaus 2003; Ozias-Akins and van Dijk 2007; Tucker and Koltunow 2009; Albertini et al. 2010; Dwivedi et al. 2010).

Regarding the determination of the megasporocyte, an analysis of mutations affecting MMC differentiation may be crucial for understanding the specification of aposporic initials during female sporogenesis. The *Arabidopsis* mutant s*porocyteless*/*nozzle* (*spl*) is unable to develop a functional MMC and shows defects in nucellar cell identity (Schiefthaler et al. 1999; Sieber et al. 2004). Moreover, mutations in the *Arabidopsis* gene *WUSCHEL* (*WUS*), a regulator of stem cell identity in the shoot apical meristem, also result in defects in MMC specification (Gross-Hardt et al. 2002). *WUS* acts by modulating the expression of *WINDHOSE1* (*WIH1*) and *WINDHOSE2* (*WIH2*), which control female sporogenesis in conjunction with *TORNADO2* (Lieber et al. 2011). Additionally, the *MULTIPLE ARCHESPORIAL CELLS1* (*MAC1*) gene of maize (Sheridan et al. 1996, 1999) and the *MULTIPLE SPOROCYTES1* (*MSP1*) and *TAPETUM DETERMINANT LIKE1A* (*TDL1A*) genes in rice (Nonomura et al. 2003; Zhao et al. 2008) are required for the production of only one megasporocyte per single ovule, indicating that these genes are involved in MMC determination. Loss of gene function results in multiple MMCs, indicating that these genes negatively regulate the sporogenous cell fate in the ovule. Zhao et al. (2002) identified the ortholog of *MSP1* in *Arabidopsis* (*EXTRA SPOROGENOUS CELLS7 EXCESS MICROSPOROCYTES1*, *EXS7EMS1*); however, its role in regulating MMC number is not known. *MSP1* encodes a leucine-rich repeat-like kinase (LRR–RLK) (Nonomura et al. 2003) that has been identified in plants as a transmembrane protein involved in a complex array of signaling pathways related to cell differentiation and developmental events (Diévart and Clark 2004).

In *Arabidopsis*, an alternative route for restricting the specification of embryo sac precursors has been proposed through the action of *ARGONAUTE* (*AGO*) genes (Olmedo-Monfil et al. 2010; Tucker et al. 2012). AGO proteins are known to be involved in post-transcriptional gene silencing mediated by short RNAs (either microRNAs or short interfering RNAs) (Baumberger and Baulcombe 2005). Small RNAs (sRNAs) have recently been studied in different model systems, and it is now known that mutations in the molecular pathways that generate sRNAs may dramatically affect fertility (Van Ex et al. 2011; Tucker et al. 2012). Previous research has demonstrated that strong mutant alleles of genes involved in the formation and activity of miRNAs, such as *AGO1*, *DCL1*, *HEN1,* and *HYL1*, disrupt reproductive development (reviewed by Van Ex et al. 2011). However, interpreting these phenotypes is frequently difficult because such mutations have ectopic effects and influence different aspects of plant development (Axtell 2013). Two members of the ARGONAUTE protein family, *AGO5* and *AGO9*, which are involved in the regulatory pathway of sRNAs in plants, have been associated with cell specification and embryo sac development (Olmedo-Monfil et al. 2010; Tucker et al. 2012). An *AGO5* ortholog in rice was reported to be essential for the progression of pre-meiotic mitosis and meiosis (Nonomura et al. 2007), and the production of viable gametes without meiosis was reported in maize lacking the ortholog of *AGO9* (Singh et al. 2011). Some of the genes isolated and characterized from sexual species may play roles in the framework of apomixis, and it is possible that sRNAs act by silencing master genes directly involved in differentiating apomictic from sexual pathways.

Particular *Arabidopsis* mutants have revealed that gametogenesis can be uncoupled from meiosis. For example, loss of certain *ARGONAUTE* (i.e., *AGO9*) genes and other genes in the small RNA pathway, such as *RNA*-*DEPENDENT RNA POLYMERASE6* (*RDR6*) and *SUPPRESSOR OF GENE SILENCING3* (*SGS3*), resulted in loss of restriction in gametic cell identity and fate in the ovule and gain of expression in multiple somatic initials in the nucellar tissue that can differentiate into gametic cells without undergoing meiosis and can initiate female gametogenesis through the activation of TEs (Olmedo-Monfil et al. 2010). Notably, these TEs are normally silenced in both developing and fully differentiated *Arabidopsis* ovules (Slotkin et al. 2009). AGO9 silences TEs in the embryo sac, and hence, its function resembles that of the PIWI (P-element-induced wimpy testis in *Drosophila*) regulatory proteins responsible for maintaining incomplete differentiation in stem cells and preserving stable cell division rates in the germ line lineage of invertebrates and mammals (Klattenhoff and Theurkauf 2008). A phenotype similar to that generated by *AGO9* loss-of-function alleles has also been observed in maize by repressing *DMT103* and *DMT102*, which are homologs of *Arabidopsis* *DOMAINS REARRANGED METHYLTRANSFERASE* (*DRM2*) and *CHROMOMETHYLASE 3* (*CMT3*) genes, respectively (Garcia-Aguilar et al. 2010). These genes are involved in new DNA methylation in *DRM2* sequences and in the retention of DNA methylation in non-CG sites in *Arabidopsis* (Cao and Jacobsen 2002). Although the function of these genes is unknown in maize, they could play a role in ensuring that a single embryo sac develops within each ovule. This mechanism could be epigenetically regulated through DNA methylation (Pillot et al. 2010a, b).

Evidence regarding the role of auxin in the cell fate specification of embryo sac development has recently been obtained. In *Arabidopsis*, two *YUCCA* (*YUC*) genes encoding proteins that are crucial for local auxin biosynthesis were shown to be expressed in the ovule, which is consistent with the role of auxin as a cell fate determinant (Pagnussat et al. 2009). Owing to the high concentrations of auxin detected in the distal tip of the nucellus at early stages of ovule development (Pagnussat et al. 2009) and because *SPOROCYTELESS*/*NOZZLE* (*SPL*) was demonstrated to repress the expression of *YUCCA* genes (Li et al. 2008), it is likely that auxin plays a key role in the cell specification machinery that regulates differentiation of the MMC and/or maintains the undifferentiated state of nucellar cells once the MMC is formed.

Proper MMC formation involves two distinct pathways: the first involves a commitment to the specification of a MMC from a somatic cell (i.e., cellular identity determination), and the second involves a commitment to the differentiation of an MMC from a single cell (i.e., cellular type specialization). Knowledge of key genes involved in both pathways should provide the molecular tools necessary for developing an artificial apomictic system in sexual plants.

In *Arabidopsis*, as in most angiosperms, the MMC undergoes regular meiosis and gives rise to a tetrad of haploid megaspores, of which three usually degenerate and one becomes the functional megaspore. Several loss-of-function phenotypes related to megasporogenesis were recently discovered in *Arabidopsis* and monocots such as rice and maize, with each showing some features of diplospory. For example, in the rice mutant *meiosis arrested at leptotene1* (*mel1*), the MMCs arrested megasporogenesis at pre-meiotic or eventually at meiotic stages (Nonomura et al. 2007). The gene controlling the *mel1* phenotype belongs to the *ARGONAUTE* (*AGO*) gene family, which is involved in several developmental processes in plants via the action of sRNAs (Vaucheret 2008). Another gene that is critical for proper megasporogenesis in *Arabidopsis* is *DYAD*/*SWITCH1* (*SWI1*), which is responsible for sister chromatid cohesion and centromere organization at meiosis. An allelic variant of this gene, *dyad*, proved to be responsible for the production of few unreduced egg cells (Ravi et al. 2008). Fully penetrant diplospory-like phenotypes were induced in *Arabidopsis* by replacing meiosis with mitosis in *MiMe* genotypes (d’Erfurth et al. 2009, 2010). In particular, these genotypes combine mutations in the two genes *SPO11*-*1*, which prevents chromosome pairing and recombination, and *REC8* (also known as *SYN1*), which modifies chromatid segregation along with *OSD1* (*MiMe*-*1* mutant; d’Erfurth et al. 2009) or *CYCA1*-*2*/*TAM* (*MiMe*-*2* mutant; d’Erfurth et al. 2010). Similar to *dyad*, these mutants were shown to form unreduced egg cells because they did not undergo a second meiotic division. Singh et al. (2011) uncovered a maize mutant, *Dominant non*-*reduction 4* (*Dnr4*), that manifested defects in chromatin condensation during meiosis with subsequent failure of chromosome complement segregation, suggesting that this mutation affects the function of chromatin remodeling factors. After substitution of a meiosis division for a mitosis-like division, these mutants formed functional unreduced egg cells that exhibited phenotypes resembling diplospory, which is similar to what was observed in the maize *elongate* (*el*1) mutant (Rhoades and Dempsey 1966) and the natural apomict *Tripsacum* (Grimanelli et al. 2003).

In maize, the *Dnr4* mutant phenotype arises due to lesions in the *AGO104* gene, which is the ortholog of *Arabidopsis* *AGO9*. Together with *MEL1*, these genes belong to the *ARGONAUTE* gene family. The abnormal patterns of cell specification mediated by the lack of AGO9 and AGO104 are reminiscent of aposporic and diplosporic apomixis, respectively, suggesting that these natural apomeiotic variants might rely on similar regulatory pathways although they are characterized by fundamental differences.

Concerning the selection of the functional megaspore, in *Arabidopsis* as in most of Angiosperms, the MMC undergoes regular meiosis and gives rise to a linear tetrad of haploid megaspores. Three of these megaspores (usually those located in the micropylar area) degenerate, and only one becomes the functional megaspore that undergoes meiosis. A distinctive process that occurs in aposporic species (e.g., *Paspalum* spp., *H. perforatum*) is the degeneration of all four megasporocytes while the already advanced aposporic embryo sacs develop further; in diplosporic species, unreduced megaspores originate via restitutional meiosis (i.e., *Taraxacum*-type, which is also widespread in *Boechera* spp.) or via a complete bypass of meiosis (i.e., *Antennaria*-type, also common in *Tripsacum dactyloides*).

In diplospory, abnormal meiosis is largely asynaptic because of the absence of pairing between homologous chromosomes and therefore results in a restitution nucleus in the first division and the formation of a dyad of unreduced megaspores (for a review, see Albertini et al. 2010). In addition to diplospory, several molecular mechanisms associated with apospory may also explain the specificity of megasporocyte degeneration, including position-dependent degeneration, cell polarization changes, and PCD (reviewed by Pupilli and Barcaccia 2012).

The easiest explanation for the occurrence of apomeiosis could be the fact that the chalazal megaspore has preferential access to nutritional elements or regulatory factors because it is closest to the maternal tissues. In aposporic ovules, the hierarchical position of the linear tetrad of megaspores could be perturbed by the growth of multiple embryo sacs that compete for nutrients and growth factors (Pupilli and Barcaccia 2012). The question remains, what happens in diplosporic ovules when meiosis is altered or bypassed? Molecular studies in *Arabidopsis* have suggested that the degeneration and death of the micropylar megaspore and the identity determination of the functional megaspore may be controlled by a positional signaling pathway (Yang and Sundaresan 2000) and/or a polarity effect, as manifested by the distribution pattern of organelles and microtubules of the cytoskeleton during megasporogenesis (Bajon et al. 1999).

Among the characteristic features of cells undergoing PCD, DNA degradation and changes in Ca^2+^ accumulation dynamics have been observed in degenerating megaspores (as reviewed by Tucker and Koltunow 2009). In lettuce, differences between Ca^2+^ accumulation pathways in the degenerating micropylar megaspore and the chalazal megaspore were observed, and the latter retained a significantly higher concentration of calcium (Qiu et al. 2008). Some members of the *MPS*-*ONE*-*BINDER* (*MOB1*) gene family in diplosporic mutants of *Medicago sativa* were specifically expressed in degenerating megaspores of normal ovules and in enlarged megaspore mother cells and embryo sacs of apomeiotic ovules (Citterio et al. 2005, 2006). *MOB1* gene products were also found in microspore tetrads at the beginning of pollen development and in the tapetum cells of anthers undergoing PCD to allow pollen dispersal at maturity. Overall, the results suggest that *MOB1* genes can play a key role in the reproductive pathway in plants. MOB proteins are involved in cell cycle progression and PCD in *Drosophila* and mammals (Hirabayashi et al. 2008; Vitulo et al. 2007).

In *Arabidopsis* and some apomictic species, one factor putatively associated with the selective degeneration of meiotic megaspores is regulated by callose boundaries and plates that can accumulate at one pole or around the MMC and in the transverse cell walls between the functional megaspore and its degenerated sister megaspores (Olmedo-Monfil et al. 2010); these boundaries and plates can also form next to the unreduced megaspore of apomeiotic dyads in *Medicago* diplosporic mutants (Albertini and Barcaccia 2007). Another factor is the *ANTIKEVORKIAN* (*AKV*) gene that, when mutated, allowed for the survival of all four megaspores in 10 % of *Arabidopsis* ovules (Yang and Sundaresan 2000). However, in *Paspalum* and other aposporic grasses, complete suppression of the sexual pathways does not always occur (Hojsgaard et al. 2008, 2013). As a consequence, different degeneration patterns of megaspores in apomictic and sexual lineages cannot be governed by specific apomixis-related genes. This process is more likely to be pleiotropically affected by a gene cascade triggering the apomictic pathway; alternatively, the sexual megaspores could be mechanically disrupted by the overgrowing aposporic embryo sacs.

Another crucial step in apomictic reproduction is the initiation and autonomous development of the embryo combined with the formation of the endosperm. Parthenogenesis, as a fertilization-independent form of embryo development, is a key component of aposporic embryo development, and it is the only feature that aposporic development has in common with diplosporic embryo development. Parthenogenesis has been widely observed in nature; however, in sexual plants, it generally occurs at a low rate in haploid egg cells (Lacadena 1974). A general rule in zygotic development is that the activation and consequent initiation of embryogenesis is trigged by the fertilization of an egg cell. In animals, egg cell fertilization induces an increase in Ca^2+^ levels at the site of sperm cell entry that propagates throughout the egg cell, inducing a downstream cascade of signals required to initiate embryogenesis (Miyazaki and Ito 2006). In plants, an increase in intracellular Ca^2+^ was observed after egg cell fertilization (Antoine et al. 2000); however, this was not sufficient to trigger parthenogenesis (Curtis and Grossniklaus 2008).

Parallel mutant screens for apomixis enabled the identification of genes controlling the fertilization-independent initiation of seed development in *Arabidopsis*. These genes, termed *FERTILIZATION*-*INDEPENDENT SEEDS* (*FIS*) genes, encode protein members of the Polycomb-related complex (Koltunow and Grossniklaus 2003). The *fis* mutants are known to initiate endosperm development without fertilization to varying extents. However, the frequency of embryo initiation by division of an egg cell is either low or does not occur at all in most *fis* mutants.

Several natural mutations are known to induce the parthenogenic development of embryos in plants when the zygote is forced to begin its growth in a haploid environment. The “Salmon” system in wheat produces high numbers of haploid parthenogenic embryos (Matzk 1996), and the *haploid inducer* (*hap*) mutant of barley is associated with parthenogenesis (Hagberg and Hagberg 1980). More recently, it was shown that parthenogenic embryos can be generated at a relatively high frequency in transgenic lines of *Arabidopsis* expressing a modified centromere-specific histone CENH3 protein (Ravi and Chan 2010). However, the question of whether these findings could be ascribable to apomictic features in wild apomicts is unclear because these mutations are related to the haploid condition of the zygote. In *Arabidopsis*, the ubiquitous overexpression of several transcription factors induces ectopic formation of embryo-like structures. *LEAFY COTYLEDON* (*LEC1* and *LEC2*) genes induce the expression of embryo-specific genes and trigger the development of embryo-like structures (Stone et al. 2001). *PICKLE* (*PKL*) is an upstream regulator of *LEC* genes that acts as repressor of embryogenesis (Henderson et al. 2004), and *BABY BOOM* (*BBM*) is an AP2-domain transcription factor that when overexpressed leads to the development of embryos and cotyledons from vegetative tissues (Boutilier et al. 2002). Moreover, the homeodomain protein encoded by *WUSCHEL* (*WUS*) is involved in promoting the vegetative to embryogenic transition and/or maintaining the identity of embryonic stem cells (Zuo et al. 2002). However, no experimental evidence supports similar functions for these genes in the egg cell or zygote. Of the genes proven to promote somatic embryogenesis in vegetative tissues, only the *Arabidopsis* ortholog of carrot *SOMATIC EMBRYOGENESIS RECEPTOR KINASE* (*SERK*) was expressed during gametogenesis and early zygotic embryogenesis (Hecht et al. 2001; Albertini et al. 2005). However, a clear phenotype could not be observed when the *SERK* gene was ectopically expressed in *Arabidopsis* (Hecht et al. 2001).

Currently, it is well known that the female gametophyte controls embryo and/or endosperm development at two different levels: (1) repression of embryo/endosperm development in the absence of fertilization through imprinting and (2) expression of factors that are required after fertilization. *FERTILIZATION*-*INDEPENDENT ENDOSPERM* (*FIE*) (Ohad et al. 1999), *MEDEA* (*MEA*) (Grossniklaus et al. 1998), and *FERTILIZATION*-*INDEPENDENT SEED2* (*FIS2*) (Chaudhury et al. 1997) repress endosperm development in the absence of fertilization. Mutations in another member of this group, *MULTICOPY SUPPRESSOR OF IRA* (*MSI1*), induce the formation of rudimentary parthenogenic embryos from growing and dividing egg cells and lead to the repressive *FIS* gene phenotype. Nevertheless, these parthenogenic embryos abort at an early stage (Guitton and Berger 2005). All these genes demonstrate homology with the Polycomb group (PcG) of genes that are involved in repressing development in *Drosophila* through chromatin remodeling mechanisms (Schwartz and Pirrotta 2007). The FIE/FIS2/MEA complex (FIS complex) acts by repressing the transcription of target genes involved directly in endosperm development. One of these genes, *PHERES1* (*PHE1*), encodes a MADS domain-containing protein. MEA and FIE proteins interact directly with the *PHE1* promoter (Köhler et al. 2003). All *fis* mutations show aberrant embryo and endosperm development if fertilized and exhibit autonomous endosperm development if unfertilized; however, the seeds abort irrespective of the paternal contribution.

Imprinting, or parent-specific expression of genes, is a mechanism by which the embryo sac controls the early stages of seed development. *MEA* was the first documented example of an imprinted gene involved in seed formation (Grossniklaus et al. 1998) in which the paternal allele is silenced and the maternal allele is expressed in the central cell. Both parental alleles are then silenced, and the modifications are removed in the central cell by a protein encoded by *DEMETER* (*DME*), a DNA glycosylase/lyase related to the DNA repair protein family (Choi et al. 2002). All genes of the *FIS* complex act through an epigenetically regulated mechanism to maintain the quiescent status of the central cell in the absence of fertilization. MULTICOPY SUPPRESSOR OF IRA (MSI1), another PcG gene product, participates together with its interacting cell cycle control protein RETINOBLASTOMA-RELATED (RBR) in the molecular machinery that represses autonomous endosperm development (Ebel et al. 2004). The MSI1/RBR complex regulates the methylation state of DNA during gametogenesis by repressing the transcription of a DNA methylase that normally controls methylation during embryo sac formation in *Arabidopsis* (Johnston et al. 2008; Jullien et al. 2008).

In plants, two main strategies have been adopted to demethylate DNA during gamete formation in ovules. One of these strategies, mediated by the MEA/DME system, is specific to the central cell, whereas the other strategy, controlled by the MSI1/RBR complex, is genomewide, affecting both the egg cell and the central cell (Sundaresan and Alandete-Saez 2010). Both mechanisms are likely to play key roles in maintaining the parent-of-origin-dependent expression of imprinted genes during gametogenesis. Therefore, it is possible that complete maternal control of reproduction (i.e., apomixis) could be achieved by modifying the epigenetic program that controls imprinting (Bicknell and Koltunow 2004). This assumption, together with the fact that down-regulation of imprinted genes in the FIS complex mimics aspects of apomictic reproduction, such as autonomous embryo and endosperm development, prompted research aimed at linking their expression to apomictic reproduction. For example, the expression profiles of FIS complex genes were proven to be similar in apomictic and sexual lines of *Hieracium* (Tucker et al. 2003). Moreover, down-regulation of the *Arabidopsis* *FIE* ortholog in *Hieracium* did not result in any autonomous endosperm development, although its activity was necessary for regular seed formation in both apomictic and sexual lines (Rodrigues et al. 2008). Similarly, gene expression studies of the *Hieracium* homolog of *Arabidopsis*, *MSI1*, in germ lineages of apomictic and sexual lines demonstrated that it is not associated with autonomous seed formation in this species (Rodrigues et al. 2010a). In fact, autonomous endosperm development was not induced in sexual species such as rice and maize by genetic transformation of *OsFIE1* (Luo et al. 2009) or by down-regulation of *ZmFIE1* and *ZmFIE2* (Rodrigues et al. 2010b). Overall, the current findings indicate that the repressive function of the FIS complex is not conserved beyond *Arabidopsis* and that FIS genes are not directly involved in triggering apomixis in sexual species.

In conclusion, factors delivered by paternal sperm cells could provide molecular cues that link egg cell fertilization to the first zygotic divisions. This scenario leads to the hypothesis that paternal–maternal triggering mechanisms could have a role in the repression of embryo development in the absence of fertilization. Moreover, epigenetic mechanisms of inactivation or down-regulation of genes by DNA methylation could represent another level of control by which the egg cell is maintained in a quiescent state in the absence of fertilization.

## Concluding remarks

Recent molecular studies aimed at understanding the basis of apomixis have failed to properly elucidate its central mystery because most apomicts are not agriculturally important crops and do not have agriculturally important relatives. Additionally, no apomicts have been sequenced so far, therefore genome annotation information is still not available. If it is true that zygotic embryogenesis (sexuality) and apomeiotic parthenogenesis (apomixis) follow similar pathways during embryo and seed development, it is also true that specific genes have to be activated, modulated, or silenced in the primary steps of plant reproduction to ensure that functioning embryo sacs develop from apomeiotic rather than meiotic cells. Other genes could be specifically or differentially expressed in sexual and apomictic plants during embryo and endosperm development.

The main approaches that have been followed to study the molecular basis of apomixis address the isolation of genes that prime the expression of apomixis in natural apomicts and/or the identification of genes that mime the features of apomictic pathways when they are deregulated in model sexual systems. To take advantage of this fundamental knowledge of apomixis with the aim of transferring it into sexual crops, three main strategies have been adopted to date: (1) the direct introgression of apomixis into crop plants by means of conventional breeding schemes; (2) the genetic transformation of crop plants by transferring exogenous genes that control the expression of apomixis; and (3) the genetic transformation of crop plants by deregulating the endogenous genes that trigger the expression of apomixis.

Although traditional breeding has taken advantage of crop species with close apomictic relatives, transferring apomixis to sexual plants has been unsuccessful to date (Savidan 2000). The introgression of apomixis into crop species from wild relatives failed mainly because natural apomicts are characterized by hybridity and polyploidy, and the loci controlling apomixis usually have a simple inheritance but a complex structure. In fact, these loci are apparently located in very large chromosomal regions, which make them recalcitrant to recombination-based genetic mapping strategies in addition to complicating their physical cloning. In the case of *Tripsacum*, the current evidence suggests the presence of barriers in the transfer of apomixis to maize (Leblanc et al. 2009). The greatest progress has been made with the introgression of apomixis into pearl millet from *Pennisetum*, where a single alien chromosome persists in the most advanced apomictic lines (Singh et al. 2010; Zeng et al. 2011). In the sexual model plant *Arabidopsis*, for each element of apomixis identified by mutation so far, the penetrance was shown to be generally low and the related candidate genes were never found to be associated with any feature of apomixis in natural apomicts. Most importantly, the three main components of apomixis could not be successfully combined into a single model plant to date. Consequently, although findings in *Arabidopsis* represent the first steps toward the synthesis of an artificial apomixis system, the engineering of asexual reproduction in *Arabidopsis* has not yet been accomplished.

Because there are a large number of candidates, it is likely that a cross-check between apomictic species should be carried out to assess both analogous and unique genes. In fact, if some of these genes are truly involved in apomixis, their functions should be conserved in other species. For example, Laspina et al. (2008) carried out a bioinformatics comparison among their own sequences and those reported by Albertini et al. (2004, 2005), identifying five genes with identical annotations. Several of the genes that are differentially expressed in both *Paspalum* spp. and *P. pratensis* appear to be involved in an ERK signal transduction cascade, with deviations controlled by a Ras ortholog and phospholipase C. Moreover, to further confirm the involvement of *APOSTART* in apomixis, partial/complete cDNA fragments showing sequence homology with this gene were isolated in *P. squamulatum*, *Cenchrus ciliaris* and *H. perforatum*. In all three species, an *APOSTART* member was found expressed specifically in parthenogenic individuals at the stage when embryo develops (Marconi et al. 2013). A similar bioinformatics comparison was also performed between the aposporic species *P. simplex* and *H. perforatum*, revealing good candidates for apomixis (located on the *ACR*, apomixis controlling region of *Paspalum*, and linked to the *HAPPY* locus of *Hypericum*) shared by these two model systems (Schallau et al. 2010; Calderini et al. 2012). In particular, this gene, known in *Arabidopsis* to be involved in the initiation of DNA replication and to be regulated transcriptionally during cell cycle, is currently under investigations in both species (Galla and Pupilli, personal communication).

We are confident that a novel perspective on the old dilemma of apomixis can emerge not only from a rigorous cross-check among apomixis candidate genes cloned in natural apomictic species but also from a high-throughput analysis of sexual mutants in which pivotal genes control the expression of apomictic components, such as apomeiosis (both apospory and diplospory), parthenogenesis, and/or autonomous endosperm development. The link between apomixis and gene-specific silencing mechanisms, including chromatin remodeling factors or trans-acting and heterochromatic small interfering RNAs involved in both transcriptional and post-transcriptional gene regulation, is beginning to become clear. In fact, merging lines of evidence regarding the role of microRNAs in the control of transcription factors, which act on genes directly involved in the development of embryo sacs, embryos, and seeds, have been reported in *Arabidopsis*.

Our opinion is that the final steps toward understanding the genetic system controlling apomixis are now being taken, and upcoming research programs will be crucial for reaching a turning point that could actually represent the “year zero” from which apomixis begins to be successfully introduced or mimed in sexual species and hence utilized in the most important crop species to support agricultural and food interests worldwide.
